# N-Acetylcysteine Administration Improves the Redox and Functional Gene Expression Levels in Spleen, Mesenteric Lymph Node and Gastrocnemius Muscle in Piglets Infected with Porcine Epidemic Diarrhea Virus

**DOI:** 10.3390/ani13020262

**Published:** 2023-01-12

**Authors:** Yanyan Zhang, Junjie Tian, Chao Wang, Tao Wu, Dan Yi, Lei Wang, Di Zhao, Yongqing Hou

**Affiliations:** Engineering Research Center of Feed Protein Resources on Agricultural By-Products, Ministry of Education, Wuhan Polytechnic University, Wuhan 430023, China

**Keywords:** piglets, porcine epidemic diarrhea virus, N-acetylcysteine, functions, spleen, mesenteric lymph node, gastrocnemius muscle

## Abstract

**Simple Summary:**

Few studies reported the effects of NAC on the lungs, liver, spleen, lymph nodes, and gastrocnemius muscles of PEDV-infected piglets. Therefore, it is very meaningful to investigate whether PEDV infection will cause adverse effects on the lung, liver, spleen, lymph node, and gastrocnemius muscle during intestinal infection of piglets, and the effects of NAC on the lung, liver, spleen, lymph node, and gastrocnemius muscle of PEDV-infected piglets. The results showed that PEDV infection has the most obvious effects on the redox and functional gene expression levels in the spleens of piglets. NAC administration ameliorated abnormal changes in measured variables in the spleens and enhanced the antioxidant capacity of the mesenteric lymph nodes and gastrocnemius muscles of PEDV-infected piglets, suggesting that NAC administration improved the redox and functional gene expression levels in the spleen, mesenteric lymph nodes, and gastrocnemius muscle in PEDV-infected piglets. This study will provide theoretical basis and technical support for the application of NAC in the prevention of PEDV infection in piglets.

**Abstract:**

Our previous study reported that N-acetylcysteine (NAC) administration improved the function of intestinal absorption in piglets infected with porcine epidemic diarrhea virus (PEDV). However, the effects of NAC administration on the functions of other tissues and organs in PEDV-infected piglets have not been reported. In this study, the effects of NAC on the liver, spleen, lung, lymph node, and gastrocnemius muscle in PEDV-infected piglets were investigated. Thirty-two 7-day-old piglets with similar body weights were randomly divided into one of four groups: Control group, NAC group, PEDV group, and PEDV+NAC group (eight replicates per group and one pig per replicate). The trial had a 2 × 2 factorial design consisting of oral administration of 0 or 25 mg/kg body weight NAC and oral administration of 0 or 1.0 × 10^4.5^ TCID_50_ PEDV. The trial lasted 12 days. All piglets were fed a milk replacer. On days 5–9 of the trial, piglets in the NAC and PEDV + NAC groups were orally administered NAC once a day; piglets in the control and PEDV groups were orally administered the same volume of saline. On day 9 of trial, piglets in the PEDV and PEDV+NAC groups were orally administrated 1.0 × 10^4.5^ TCID_50_ PEDV, and the piglets in the control and NAC groups were orally administrated the same volume of saline. On day 12 of trial, samples, including of the liver, spleen, lung, lymph node, and gastrocnemius muscle, were collected. PEDV infection significantly increased catalase activity but significantly decreased the mRNA levels of *Keap1*, *Nrf2*, *HMOX2*, *IFN-α*, *MX1*, *IL-10*, *TNF-α*, *S100A12*, *MMP3*, *MMP13*, *TGF-β*, and *GJA1* in the spleens of piglets. NAC administration ameliorated abnormal changes in measured variables in the spleens of PEDV-infected piglets. In addition, NAC administration also enhanced the antioxidant capacity of the mesenteric lymph nodes and gastrocnemius muscles in PEDV-infected piglets. Collectively, these novel results revealed that NAC administration improved the redox and functional gene expression levels in the spleen, mesenteric lymph nodes, and gastrocnemius muscle in PEDV-infected piglets.

## 1. Introduction

The pig industry accounts for a large proportion of animal farming in China [1,2]. Piglet feeding can determine the slaughter time, slaughter quantity, and meat quality of finishing pigs [3]. However, because the immune and digestive systems in piglets are not fully developed, the disease resistance of newborn piglets is very fragile [4]. Once piglets are infected with virus and bacterium, it will lead to poor growth and even death in piglets.

Porcine epidemic diarrhea (PED) is an acute highly contagious intestinal infectious disease caused by porcine epidemic diarrhea virus (PEDV) [5]. In recent years, a high-incidence trend has been shown in PED, causing heavy losses to some large-scale pig farms [6]. PEDV infection leads to a very high mortality rate of suckling piglets, which seriously restricts the healthy development of the pig industry [7]. PEDV is mainly transmitted through the “fecal–oral” route, but also through water sources, feed, vehicles, or pens [8]. The latest study found that PEDV could also be transmitted through the nasal cavity [9]. Clinical symptoms caused by PEDV are generally characterized by severe watery diarrhea, thin yellow or grayish yellow stools, dehydration, and vomiting. Pigs infected with PEDV showed mental depression, sunken eye sockets, and loss of appetite. Sick pigs died of severe dehydration 3 to 4 days after diarrhea. Pigs of different ages are susceptible to PEDV, and PEDV is the most harmful to newborn piglets. PEDV often infects whole litters, with mortality close to 100% [10,11,12,13]. Piglets infected with PEDV are usually accompanied by the infection of *Escherichia coli*, *Streptococcus*, porcine circovirus, and porcine rotavirus. The mixed infection leads to a very complicated etiology of diarrhea in piglets, which increases the difficulty of prevention and the mortality of piglets. At present, prevention strategies are mainly based on the pathogenic biology of PEDV around the world [14,15,16]. However, due to the wide variety of viruses and diverse pathogenesis, the effectiveness of prevention strategies is limited. At present, PEDV infection is mainly prevented by injection of PEDV attenuated vaccine and inactivated vaccine in China [17]. However, the mucosal immunity induced by the vaccine is limited, and the virus continues to mutate, making the existing vaccine less and less effective in preventing PEDV [18]. Therefore, effective prevention strategies need to be further studied.

N-acetylcysteine (NAC), an amino acid derived from L-cysteine, is one of the important biologically active substances in the body [19,20]. Since cysteine has a pungent odor, cysteine is acetylated to reduce the pungent odor, which also makes its structure more stable [21]. Therefore, NAC is widely used as a derivative of cysteine. The chemical formula of NAC is C_5_H_9_NO_3_S, and the molecular weight is 163.2. NAC is a small molecule with active sulfhydryl side chain and antioxidant activity. The popularity and widespread use of NAC are partly due to its easy availability [22]. A previous study has shown that trace amounts of NAC can be found in eukaryotes, including many tissues and organs [23]. Allium plants, especially vegetables such as onions, are rich in organic sulfur compounds in our daily diet and serve as an important source of NAC intake [24]. 

As a small molecule, NAC is easily absorbed by the intestine. A large number of studies have found that NAC can promote the synthesis of glutathione (GSH), which is the most important non-enzymatic antioxidant substance in the body through deacetylation, indicating that NAC could enhance the body’s antioxidant capacity [11,19,20]. Due to the existence of sulfhydryl side chain, GSH has strong antioxidant capacity, which can interfere with the generation of free radicals in the body, eliminate the generated free radicals, regulate cell metabolism and gene expression and signal transduction system, resist apoptosis, and prevent DNA damage [25]. Studies have shown that NAC can regulate the expression of nuclear factor-kappa B (NF-κB), which is involved in the transcriptional regulation of immune and inflammatory response-related genes [26]. In addition, NAC can also inhibit virus replication, stimulate the immune response of T-lymph node cells, and strengthen the adhesion and phagocytosis of macrophages, thereby protecting cells from damage caused by virus infection [27].

As early as the 1960s, NAC was studied by a large number of medical researchers, and it was clinically used to treat respiratory system-related diseases with anti-oxidative and anti-inflammatory effects [28]. Previous study has reported that NAC can effectively treat liver diseases such as acute liver failure, chronic liver failure, and liver failure caused by alcoholism [29]. Meanwhile, NAC, as a donor liver preservation solution in liver transplantation, can significantly reduce the mortality and morbidity of high-risk patients, and it is a very promising direction for NAC research [30]. In the treatment of lung diseases, NAC can not only stimulate alveolar epithelial cells to secrete surfactant and then expand the alveoli and increase gas exchange area, but also protect alveolar elasticity and ensure breathing by activating anti-protease and inhibiting the release of elastase from polymorphonuclear neutrophils [31,32]. In the treatment of cerebral ischemia-reperfusion injury, NAC can downregulate the activity of inflammatory cells and has a protective effect on spleen after cerebral ischemia reperfusion [33]. NAC, as a strong antioxidant, may protect ovarian function by improving the redox balance when treating PCOS β cells [34]. Therefore, NAC has multiple functions.

Previous study reported that PEDV can acutely infect villous epithelial cells of piglet intestine and cause acute, severe atrophic enteritis [35]. The villous in PEDV-infected small intestine is marked atrophy or fusion due to acute necrosis and exfoliation from the lamina propria in small intestinal villous enterocytes, which causes profound vomiting and diarrhea and is a main cause of nursing piglet death [7,11,35]. As PEDV mainly causes intestinal injury in piglets, current studies are mainly focused on nutrient regulators that can effectively improve intestinal injury in PEDV-infected piglets [11,36,37]. Our laboratory has established an intestinal injury model of a PEDV-infected piglet and confirmed that NAC can effectively alleviate the intestinal injury caused by PEDV infection by reducing oxidative stress and the expression of MX1, improving substance transport and inhibiting inflammatory responses [11,36]. However, it is not clear whether NAC can also exert beneficial effects on the lung, liver, spleen, lymph node, and gastrocnemius in PEDV-infected piglets. In addition, the intestinal injury of PEDV-infected piglet intestine is mainly studied; few studies reported whether PEDV infection also has adverse effects on the lungs, liver, spleen, lymph nodes, and gastrocnemius muscles of piglets. Therefore, it is very meaningful to investigate whether PEDV infection will cause adverse effects on the lung, liver, spleen, lymph node, and gastrocnemius muscle during intestinal infection of piglets; and the effects of NAC on the lung, liver, spleen, lymph node, and gastrocnemius muscle of PEDV-infected piglets, which will provide theoretical basis and technical support for the application of NAC in the prevention of PEDV infection in piglets. In view of the antioxidant and anti-inflammatory capacity of NAC and its functions in substance transport and regulation of interferon signaling pathway related genes and so on [11,19,26,28,29,30,32,33,34,36], this study investigated the effects of NAC on the redox and functional gene expression levels in the spleen, mesenteric lymph nodes, and gastrocnemius muscle in PEDV-infected piglets.

## 2. Materials and Methods

### 2.1. Animal Care and Diets

An animal trial was approved by the Institutional Animal Care and Use Committee at Wuhan Polytechnic University (no. WPU202001001). 

Thirty-two 7-day-old crossbred female piglets (Duroc × Landrace × Yorkshire, similar initial weight) were purchased from a porcine reproductive and respiratory syndrome virus (PRRSV), African swine fever virus (ASFV), porcine epidemic diarrhea virus (PEDV), and porcine circovirus 2 (PCV 2)-negative commercial breeding farm. The management and feeding of piglets were conducted as previously described with modifications [38]. During trials, the piglets were housed individually in pens (2 × 3 m^2^), and the room temperature was maintained at 26–28 ℃. The basal diet used in this experiment was liquid milk replacer (Table 1), which could meet all the nutritional needs of sucking piglets. The milk replacer (powder), which was purchased from Wuhan Anyou Feed Co., Ltd (Wuhan, China), was dissolved in warm water (50–60 °C) to form a liquid feed prior to feeding. The piglets were fed 5 times a day (8:00, 12:00, 15:00, 18:00, 21:00), and each piglet was fed 20 g of a milk replacer (powder) per meal. The warm water was fed three times a day (8:00, 15:00, 21:00). The feed intake of the piglets was observed after each feeding.

### 2.2. Experimental Design

The trial lasted for 12 days. During the trial, all piglets were fed the same basic diet (milk replacer). On days 5–9 of trial, piglets in the NAC group and PEDV+NAC group were orally administrated NAC (25 mg/kg BW, NAC purchased from Sigma Chemical was dissolved with sterile saline) once a day. Piglets in the control and PEDV groups were orally administrated the same volume of sterile saline. On day 9 of trial, piglets in the PEDV and PEDV+NAC groups were orally administrated PEDV (1.0 × 10^4.5^ TCID_50_ per piglet, PEDV Yunnan strain) [11]. Piglets in the control and NAC groups were orally administrated the same volume of sterile saline. The trial was arranged in a 2 × 2 factorial design after PEDV infection. The main factors consisted of oral administration of NAC (0 or 25 mg/kg body weight) and oral administration of PEDV (0 or 1.0 × 10^4.5^ TCID_50_). The doses of PEDV and NAC were chosen according with our previous study [11]. On day 12 of the trial, all piglets were slaughtered under anesthesia (sodium pentobarbital, 50 mg/kg BW, iv) to collect the samples.

### 2.3. Samples Collection

On day 12 of the trial, the same tissue parts of the livers, spleens, and lungs were collected. The liver tissues with a length of 3 cm and a width of 2 cm were from the middle part of the right lobe edge of the liver. The intact spleen tissue was collected. The lung tissues with a length of 3 cm and a width of 1 cm were from the middle lobe of the right lung. The intact mesenteric lymph nodes and part of the gastrocnemius muscle (3 cm from the middle part) were collected. The collected samples were immediately frozen in liquid nitrogen and stored at −80 °C until analysis. All samples were collected within 15 min after dissecting.

### 2.4. The Determination of the Levels of Oxidative Enzymes and Oxidation-Relevant Products in the Liver, Spleen, Lung, Lymph Node, and Gastrocnemius Muscle

The liver, spleen, lung, lymph node, and gastrocnemius muscle were used for analyses of oxidative enzymes and oxidation products. The activities of catalase (CAT), glutathione peroxidase (GSH-Px), Myeloperoxidase (MPO), and superoxide dismutase (SOD); and the concentrations of hydrogen peroxide (H_2_O_2_) and malondialdehyde (MDA), were determined as described by Wang et al. [11]. Catalase (CAT), glutathione peroxidase (GSH-Px), myeloperoxidase (MPO), total superoxide dismutase (T-SOD), hydrogen peroxide (H_2_O_2_), and malondialdehyde (MDA) in the intestinal mucosae were determined by using commercially available kits, including the catalase (CAT) assay kit (Ultraviolet, A007-2-1), glutathione peroxidase (GSH-PX) assay kit (Colorimetric method, A005-1-2), myeloperoxidase assay kit (A044-1-1), total superoxide dismutase (T-SOD) assay kit (Hydroxylamine method, A001-1-2), hydrogen peroxide assay kit (A064-1-1), and malondialdehyde (MDA) assay kit (TBA method, A003-1-2) (Nanjing Jiancheng Bioengineering Institute, Nanjing, China).

### 2.5. Realtime Fluorescence Quantitative PCR (qPCR)

To quantify the mRNA levels of Kelch-1ike ECH-associated protein l (*Keap1*), NF-E2-related factor 2 (*Nrf2*), glutathione S-transferase omega 2 (*GSTO2*), heme oxygenase 1 (*HMOX1*), matrix metallopeptidase 3 (*MMP3*), matrix metalloproteinase 13 (*MMP13*), transforming growth factor-β (*TGF-β*), regenerating islet-derived 3 gamma (*REG3G*), gap junction protein alpha 1 (*GJA1*), interleukin-1 beta (*IL-1β*), interleukin-4 (*IL-4*), interleukin-6 (*IL-6*), interleukin-10 (*IL-10*), tumor necrosis factor-α (*TNF-α*), S100 calcium binding protein A12 (*S100A12*), transient receptor potential channel subfamily V member 6 (*TRPV6*), potassium voltage-gated channel subfamily J member 13 (*KCNJ13*), interferon-α (*IFN-α*), mucovirus resistance protein 1 (*MX1*), 2′-5′-oligoadenylate synthetase-like (*OASL*), interferon stimulated gene 15 (*ISG15*), and guanylate binding protein 2 (*GBP2*) in the liver, spleen, lung, lymph node, and gastrocnemius muscle, the total mRNA in different tissues was extracted as previously described with some modifications [39]. Briefly, the frozen tissue samples (100 mg) were powdered after nitrogen freezing. The total mRNA in different tissues was isolated using the RNAiso Plus kit according to the manufacturer’s instructions. The concentrations and purities of mRNA were determined by using NanoDrop^®^ ND-1000A UV-vis spectrophotometer (Thermo Scientific, Wilmington, DE, USA) and 1% denatured agarose gel electrophoresis. cDNA was synthesized by using a PrimeScript^®^ RT reagent kit with gDNA Eraser (Takara, Dalian, China) according to the manufacturer’s instructions and stored at −80 °C until use.

The qPCR was used to analyze the gene expression in different tissues. The primers used in this study are shown in Table 2. RPL4 was used as the reference gene. The levels of gene expression in different tissues were determined by using the SYBR^®^ Premix Ex Taq^TM^ on the applied biosystems 7500 fast real-time PCR system (Foster City, CA, USA). Results of the qPCR reactions (50 µL) were analyzed by using the melting curves of the products, as described by the previous study, and the 2^−ΔΔCt^ method, as described by the previous study [39]. Each biological sample was run in triplicate.

### 2.6. Statistical Analysis

The data for the redox and mRNA levels are expressed as means with pooled standard error of mean (SEM) and analyzed by using SPSS 20.0 (SPSS Inc. Chicago, IL, USA), appropriate for a 2 × 2 factorial arrangement with NAC and PEDV as the main effects. The differences between treatment means were determined by the Duncan’s multiple range test. *p*-values < 0.05 were taken to indicate statistical significance. A *p*-value < 0.10 but >0.05 was considered as a trend. 

## 3. Results

### 3.1. The Levels of Redox Status in Liver, Spleen, Lung, Lymph Node, and Gastrocnemius Muscle

Data on the activities of CAT, MPO, SOD, and GSH-Px, and the concentrations of H_2_O_2_ and MDA, are summarized in Table 3. Compared with non-infected (−PEDV) piglets, PEDV-infected piglets had lower CAT activities in the lungs and lymph nodes; lower MPO, SOD, and GSH-Px activities in the spleen; lower H_2_O_2_ concentrations in the lungs and spleen; higher MDA concentrations in the lungs and lymph nodes; higher CAT activities in gastrocnemii and spleen; higher MPO activity in the lymph nodes; higher GSH-Px activity in the lungs; and a higher MDA concentration in the gastrocnemii. There were PEDV × NAC interactions in CAT activities; high MPO concentrations in the gastrocnemii, lymph nodes, and spleen; high SOD and GSH-Px activities and H_2_O_2_ concentration in lymph nodes; high GSH-Px activity and MDA concentration in the gastrocnemii; and high H_2_O_2_ concentration in the lungs. The data indicated NAC administration could increase CAT and GSH-Px activities and MDA concentration in the gastrocnemii, and MPO and SOD activities and H_2_O_2_ concentration in lymph nodes; and reduce CAT activity in spleen and H_2_O_2_ concentration in the lungs in PEDV-infected (+PEDV) piglets. NAC administration increased CAT activities in the gastrocnemii and spleen, and reduced CAT and SOD activities in the lymph nodes, MPO activity in spleen, and GSH-Px activity in the gastrocnemii in non-infected (−PEDV) piglets. There were no PEDV and NAC interactions in CAT activity in the liver and MDA concentrations in lung. However, the piglets administrated NAC had a higher CAT activity in liver, and a lower MDA concentration in lung. Specifically, NAC administration attenuated the elevations of CAT activity in spleen and reductions of SOD activity in lymph nodes and GSH-Px activity in the gastrocnemii in the PEDV-infected piglets.

### 3.2. The mRNA Levels of Keap1, Nrf2, GSTO2, and HMOX1 in Liver, Spleen, Lung, Lymph Node, and Gastrocnemius Muscle

Data on *Keap1*, *Nrf2*, *GSTO2*, and *HMOX1* mRNA levels are summarized in Table 4. Compared with non-infected (−PEDV) piglets, PEDV-infected piglets had higher *Keap1* mRNA levels in the lungs and lymph nodes; higher *Nrf2* mRNA levels in the gastrocnemii and spleen and *HMOX1* mRNA levels in the lymph nodes and spleen; a lower *Nrf2* mRNA level in liver; lower *GSTO2* mRNA levels in liver, lymph nodes, gastrocnemii, and spleen; and a lower *HMOX1* mRNA level in liver. There were PEDV × NAC interactions in *Keap1* mRNA levels in lung and spleen; *Nrf2* mRNA levels in liver, lungs, gastrocnemii, and spleen; *GSTO2* mRNA levels in liver, lymph nodes, gastrocnemii, and spleen; and *HMOX1* mRNA levels in the liver, lungs, and spleen.

Our results also showed that NAC administration increased the *Keap1* mRNA level in the lungs; increased the *Keap1*, *Nrf2*, and *HMOX1* mRNA levels in spleen; and reduced *Nrf2* mRNA levels in the liver and gastrocnemii in PEDV-infected (+PEDV) piglets. NAC administration increased *Nrf2* mRNA levels in liver and lung and the *HMOX1* mRNA level in liver, and reduced *Nrf2* and *HMOX1* mRNA levels in spleen, and *GSTO2* mRNA levels in liver, lymph nodes, and gastrocnemii in non-infected (−PEDV) piglets. No PEDV × NAC interaction was observed for the *Nrf2* mRNA level in lymph nodes, the *GSTO2* mRNA level in lungs, and *HMOX1* mRNA levels in lymph nodes and gastrocnemii samples. However, NAC administration increased the *HMOX1* mRNA level in lymph nodes; and reduced the *Nrf2* mRNA level in lymph nodes, the *GSTO2* mRNA level in the lungs, and the *HMOX1* mRNA level in the gastrocnemii. Specifically, NAC administration attenuated the reductions of mRNA levels for *Keap1* in the lungs and spleen, and *Nrf2* and *HMOX1* in spleen, and the elevation of the *Nrf2* mRNA level in the gastrocnemii in the PEDV-infected piglets.

### 3.3. IFN-α, MX1, OASL, and ISG15 mRNA Levels in Liver, Spleen, Lung, Lymph Node, and Gastrocnemius Muscle

Data on *IFN-α*, *MX1*, *OASL*, and *ISG15* mRNA levels are summarized in Table 5. Compared with non-infected (−PEDV) piglets, PEDV-infected piglets had higher *IFN-α* mRNA levels in the spleen and gastrocnemii; higher *MX1* and *ISG15* mRNA levels in the liver, lymph nodes, and gastrocnemii; higher *OASL* mRNA levels in the lymph nodes and gastrocnemii; a reduced *IFN-α* mRNA level in the liver and *MX1*; and reduced *ISG15* mRNA levels in the spleen. There were PEDV × NAC interactions in IFN-α mRNA levels in the liver, spleen, and lymph node; *MX1* mRNA levels in the liver, spleen, and gastrocnemii; and *OASL* and *ISG15* mRNA levels in the spleen, lymph node, and gastrocnemii. 

Our results also showed that NAC administration increased *IFN-α*, *MX1*, *OASL*, and *ISG15* mRNA levels in spleen tissue; increased *IFN-α*, *OASL*, and *ISG15* mRNA levels in lymph nodes; and reduced *MX1*, *OASL*, and *ISG15* mRNA levels in the gastrocnemii in PEDV-infected (+PEDV) piglets. NAC administration increased *IFN-α* and *MX1* mRNA levels in the liver, the *IFN-α* mRNA level in lymph nodes, and the *ISG15* mRNA level in the spleen; and reduced *IFN-α* and *OASL* mRNA levels in the spleen in non-infected (−PEDV) piglets. No PEDV × NAC interaction was observed for the *MX1* mRNA level in lymph nodes. However, NAC administration increased the *MX1* mRNA level in lymph node. Specifically, NAC administration attenuated the reductions of mRNA levels for *IFN-α*, *MX1*, and *OASL* in the spleen; and the elevations of mRNA levels for *MX1*, *OASL*, and *ISG15* in the gastrocnemii in the PEDV-infected piglets.

### 3.4. TRPV6 and KCNJ13 mRNA Levels in Liver, Spleen, Lung, Lymph Node, and Gastrocnemius Muscle

Data on *TRPV6* and *KCNJ13* mRNA levels are summarized inTable 6. Compared with non-infected (−PEDV) piglets, PEDV-infected piglets had higher *TRPV6* mRNA levels in gastrocnemii; higher *KCNJ13* mRNA levels in the spleen, lymph node, and gastrocnemii; lower *TRPV6* mRNA levels in the liver and lung; a lower and *KCNJ13* mRNA level in the liver. There were PEDV × NAC interactions in *TRPV6* mRNA levels in liver and lung and *KCNJ13* mRNA levels in the liver and gastrocnemii. Our results also showed that NAC administration reduced *TRPV6* and *KCNJ13* mRNA levels in the liver and increased the *KCNJ13* mRNA level in the gastrocnemii in PEDV-infected (+PEDV) piglets. NAC administration increased *TRPV6* mRNA levels in the liver and lung and the *KCNJ13* mRNA level in the liver in non-infected (−PEDV) piglets. No PEDV × NAC interaction was observed for the *TRPV6* mRNA level in lymph nodes or the *KCNJ13* mRNA levels in the spleen and lymph nodes. However, NAC administration increased the *TRPV6* mRNA level in the lymph node and *KCNJ13* mRNA levels in the spleen and lymph node.

### 3.5. The mRNA Levels of IL-1β, IL-4, IL-10, TNF-α, and S100A12 in Liver, Spleen, Lung, Lymph Node, and Gastrocnemius Muscle

Data on *IL-1β*, *IL-4*, *IL-10*, *TNF-α*, and *S100A12* mRNA levels are summarized in Table 7. Compared with non-infected (−PEDV) piglets, PEDV-infected piglets had a higher *IL-4* mRNA level in the lungs and higher *IL-10* and *S100A12* mRNA levels in lymph nodes; and lower *IL-1β* mRNA levels in the liver, lymph nodes, and gastrocnemii. They had lower *IL-4* mRNA levels in lymph nodes, *IL-10* mRNA levels in liver, lung, and spleen; and *TNF-α* mRNA levels in liver and gastrocnemius. There were PEDV × NAC interactions in *IL-10* mRNA levels in liver, lung, lymph node, and spleen; *IL-4* mRNA levels in lung and lymph node; *IL-10* mRNA levels in spleen and lymph node; the *TNF-α* mRNA level in spleen; and *S100A12* mRNA levels in liver, lung, spleen, and lymph node. Our results show that NAC administration increased the *IL-4* mRNA level in lymph nodes; and the *IL-10*, and *TNF-α*, and *S100A12* mRNA levels in the spleen. It also reduced the *IL-1β* mRNA level in lymph nodes and the *S100A12* mRNA level in liver in PEDV-infected (+PEDV) piglets. NAC administration increased the *IL-1β* mRNA levels in liver and lymph nodes, the *IL-4* mRNA level in the lungs, the *TNF-α* mRNA level in liver, and the *S100A12* mRNA levels in liver and spleen; and reduced *IL-1β* mRNA levels in spleen and gastrocnemius, the *IL-4* mRNA level in lymph nodes, *IL-10* mRNA levels in spleen and gastrocnemius, the *TNF-α* mRNA level in spleen, and the *S100A12* mRNA level in spleen in non-infected (−PEDV) piglets. No PEDV × NAC interaction was observed for the *IL-10* mRNA level in lung or *TNF-α* mRNA levels in liver and gastrocnemius. However, NAC administration increased the *IL-10* mRNA level in lung and the *TNF-α* mRNA level in liver; and reduced the *TNF-α* mRNA level in gastrocnemius. Specifically, NAC administration attenuated the reductions in mRNA levels of *IL-10*, *TNF-α*, and *S100A12* in spleen and *IL-4* in lymph nodes; and the elevation in the mRNA level of *S100A12* in liver in the PEDV-infected piglets.

### 3.6. The mRNA levels of MMP3, MMP13, REG3G, TGF-β, and GJA1 in liver, spleen, lung, lymph node, and gastrocnemius muscle

Data on *MMP3*, *MMP13*, *REG3G*, *TGF-β*, and *GJA1* mRNA levels are summarized in Table 8. Compared with non-infected (−PEDV) piglets, PEDV-infected piglets had higher *MMP3* mRNA levels in the lungs, spleen, and lymph nodes; *MMP13* mRNA levels in the lungs and gastrocnemii; and *REG3G* mRNA levels in the spleen and gastrocnemii. They also had a lower *MMP3* mRNA level in the gastrocnemii, *MMP13* mRNA level in the spleen, *TGF-β* mRNA levels in the liver and gastrocnemius, and *GJA1* mRNA levels in the lungs and gastrocnemii. There were PEDV × NAC interactions in *MMP3*, *MMP13*, and *TGF-β* mRNA levels in the spleen and lymph nodes; *TGF-β* and *MMP13* mRNA levels in the liver; *REG3G* mRNA levels in the spleen and gastrocnemius; and *GJA1* mRNA levels in the liver and spleen. Our results also showed that NAC administration increased the mRNA levels of *MMP3*, *MMP13*, *REG3G*, *TGF-β*, and *GJA1* in spleen; the *MMP13* mRNA level in gastrocnemii; and the *TGF-β* mRNA level in lymph nodes. It reduced *MMP3* mRNA levels in lymph nodes and gastrocnemii, the *REG3G* mRNA level in gastrocnemii, and the *GJA1* mRNA level in liver in PEDV-infected (+PEDV) piglets. NAC administration increased the *MMP3* mRNA levels in lymph nodes and gastrocnemii, *MMP13* mRNA levels in the liver and lymph nodes, the *REG3G* mRNA level in spleen, and the *GJA1* mRNA level in the liver; and reduced the *MMP3* mRNA level in the spleen and *TGF-β* mRNA levels in the spleen and gastrocnemius in non-infected (−PEDV) piglets. No PEDV × NAC interaction was observed for *TGF-β* mRNA levels in the liver or lungs, or *GJA1* mRNA level in gastrocnemii. However, NAC administration increased the *TGF-β* mRNA level in liver and the *GJA1* mRNA level in gastrocnemii, and reduced the *TGF-β* mRNA level in the lungs. Specifically, NAC administration attenuated the reductions in mRNA levels of *MMP3*, *MMP13*, *TGF-β*, and *GJA1* in the spleen and *TGF-β* in lymph nodes, and the elevations of mRNA levels of *MMP3* in lymph nodes and gastrocnemii in the PEDV-infected piglets.

## 4. Discussion

Based on the previous finding that dietary NAC administration not only improved the intestinal absorptive function, but also alleviated intestinal injury in PEDV-infected piglets [11,37], this study investigated the effects of NAC on the functions of the liver, spleen, lung, gastrocnemius, and lymph node in PEDV-infected piglets. Currently, PEDV has been widely studied as a virus that can colonize the intestines of piglets. It can damage the intestinal barrier structure in piglets, resulting in loss of appetite and severe diarrhea [12,40]. So far, PEDV has brought huge economic losses to the pig industry [7,10]. However, there are few studies on the effects of PEDV on the functions of the liver, spleen, lung, gastrocnemius, and lymph node in piglets. In this study, the redox and functional gene expression levels in the liver, spleen, lung, gastrocnemius, and lymph node in PEDV-infected piglets were investigated. These results showed that the changes in the redox and functional gene expression levels in the spleen in PEDV-infected piglets were the most obvious. PEDV infection significantly decreased the activities of SOD and GSH-Px, and the mRNA levels of 16 genes (*Keap1*, *Nrf2*, *GSTO2*, *HMOX1*, *IFN-α*, *MX1*, *OASL*, *ISG15*, *IL-1β*, *IL-10*, *TNF-α*, *S100A12*, *MMP3*, *MMP13*, *TGF-β*, and *GJA1*) in spleens of piglets. The activities of CAT and SOD, and the mRNA levels of five genes (*Nrf2*, *GSTO2*, *IL-1β*, *IL-4*, and *TGF-β*) in the lymph nodes in PEDV-infected piglets were decreased. The activity of GSH-Px and the mRNA levels of six genes (*GSTO2*, *IL-1β*, *IL-10*, *TNF-α*, *TGF-β*, and *GJA1*) in the gastrocnemii of PEDV-infected piglets were decreased. The mRNA levels of three genes (*IL-10*, *TNF-α*, and *TGF-β*) in the liver in PEDV-infected piglets were decreased. PEDV infection had little effect on the redox level of the lungs in piglets; only decreased activity of CAT was detected. These results revealed that PEDV infection not only can cause intestinal injury, but also can downregulate the redox state and mRNA levels of multiple functional genes in the spleen, lymph node, gastrocnemius, liver, and lung in piglets. Among them, the reduction in redox level and downregulation of genes were the greatest in the spleen—followed by the lymph nodes, gastrocnemii, and liver, and the least in the lungs—after PEDV infection. Therefore, it is of guiding significance to study the effects of NAC on the redox and functional gene expression levels of the spleen in piglets infected with PEDV.

The oxygen metabolism process in the body cells will produce a large number of reactive oxygen species and reactive nitrogen substances, such as common superoxide anions, hydrogen peroxide, hydroxyl radicals, nitric oxide, nitrogen dioxide, and other free-radical ion groups [41]. Under normal circumstances, the internal oxidation and antioxidant capacity of the body will maintain a dynamic balance. The imbalance of the oxidation system and antioxidant system induced by internal and external environmental factors will cause a large amount of oxidative free radicals (ROS) to accumulate [42]. If the accumulated ROS in the body cannot be cleared in time, oxidative stress will occur, which may lead to the damage of biological macromolecules and the abnormality of cell structure and function, eventually leading to increased morbidity and mortality in piglets [43]. An antioxidant reaction is a kind of self-protection function of organisms from free radical damage, in which antioxidant enzymes can enhance the body’s defense and immune ability, and its activity can reflect the body’s oxidation antioxidant status [44]. Studies have shown that the appropriate concentrations of reactive oxygen species, which are indispensable in many metabolic processes, can help the body resist some harmful bacteria and assist cell signal transduction [45,46]. Studies have also shown that the content of free radicals in the body and the nutritional status of the body under normal physiological conditions maintain a dynamic balance [47]. Once this balance is broken, the production of free radicals in the body will be increased, causing oxidative damage to the body’s cells [48]. SOD is a kind of active protease existing in animal body, which can scavenge oxygen free radicals autonomously and convert oxygen free radicals into H_2_O_2_ by disproportionation reaction [49]. Subsequently, CAT converts H_2_O_2_ into water, thereby reducing the damage caused by H_2_O_2_ [50]. In general, the activity of SOD is used as the standard to judge the body’s ability to scavenge oxygen free radicals [51]. In addition, GSH-Px is another kind of antioxidant enzyme that can transform peroxide into non-toxic hydroxyl compounds through a reduction reaction and plays an important role in maintaining the integrity of the cell membrane structure [49]. MPO is an iron-containing lysosome, which is closely related to the functions and activation of neutrophils [39]. MDA is a product of lipid peroxidation, and its activity can indirectly reflect the degree of host damage, especially the degree of cell membrane peroxidation [51]. However, PEDV infection significantly weakened the antioxidant capacity of the spleen, lymph node, and gastrocnemius by decreasing the activities of SOD (spleen and lymph node), GSH-Px (spleen and gastrocnemius), and CAT (lymph node). NAC administration did not change the weakened antioxidant capacity of the spleen, lymph nodes, and gastrocnemius in piglets. These results revealed that NAC administration cannot alter the reduction in antioxidant enzyme activities caused by PEDV infection.

The Keap1-Nrf2/ARE signaling pathway participates in and regulates cellular antioxidant stress. This signaling pathway can regulate the mRNA levels of downstream antioxidant genes to make the body return to a normal physiological state. The mRNA levels of *Keap1* and *Nrf2* can reflect the ability of cells to scavenge free radicals and lipid peroxidation damage [52]. GSTs, as catalysts, can regulate electrophilic reagents, carcinogens, genotoxic xenobiotics, and cytotoxic xenobiotics through reduced glutathione, so that these external stimulants can be transformed into hydrophilic and easily excreted substances, which can be excreted from the body, thereby reducing the oxidative stress caused by these external stimulants [53]. GSTO2, as an important subtype of GSTs, can regulate the activities of antioxidant enzymes by changing the transcription levels of related antioxidant genes and reduce the negative effects of oxidative stress [54]. HMOX1 is the rate-limiting enzyme that catalyzes the oxidation of heme to biliverdin, carbon monoxide, and free iron in the heme degradation pathway. HMOX1, also known as heat shock protein 32, can play an important role in many kinds of stimulation and pathological states, such as high temperature, hypoxia, oxidative stress, apoptosis, and mucosal damage [55]. However, PEDV infection significantly reduced the mRNA levels of *Keap1* (lung and spleen), *Nrf2* (lymph node and spleen), *GSTO2* (liver, lymph node, spleen, and gastrocnemius), and *HMOX1* (liver and spleen). NAC administration significantly decreased the reductions in the mRNA levels of *Keap1* (lung and spleen), *Nrf2* (spleen), and *HMOX1* (spleen). These results revealed that NAC administration can improve the antioxidant levels of the spleen and lung by up-regulating the expression levels of antioxidant-related genes in PEDV-infected piglets.

IFN-α is a glycoprotein with multiple functions produced by monocyte macrophages, dendritic cells, and B cells. It has a significant immunomodulatory effect, has shown anti-tumor and anti-virus activity, and plays a key role in the body’s immune defense [56]. A study has shown that the expression of Mx1 is positively correlated with the secretion of IFN-α and IFN-β. Type-I interferon (including IFN-α and IFN-β) has an important role in the host’s anti-viral response. It has biological activities such as anti-viral activity, regulating host immunity, and anti-tumor activity [57]. However, part of its anti-viral process requires the help of various biological activity genes. Mx1 may act as intracellular mediator precisely in the antiviral process induced by type-I interferon [58]. ISG15 is also a protein highly expressed by mammalian cells after being stimulated by interferon and virus. OASL is a protein with broad-spectrum antiviral activity and is induced by type-I interferon. It is expressed in almost all vertebrates and has antiviral effects [59]. In this study, the mRNA levels of *IFN-α* (liver and spleen), *MX1* (spleen), and *OASL* (spleen) were significantly decreased in piglets infected with PEDV. PEDV administration significantly decreased the reductions in the mRNA levels of *IFN-α*, *MX1*, and *OASL* in the spleen in PEDV-infected piglets. In addition, NAC administration significantly increased the mRNA level of *ISG15* in the spleens of PEDV-infected (+PEDV) piglets. These results suggested that NAC administration resisted the infection of PEDV by regulating the expression levels of immune-related genes in the spleen.

TRPV6, as a member of the transient receptor potential channel subfamily, is a highly selective Ca^2+^ transmembrane transport channel, which is mainly responsible for the active Ca^2+^ transmembrane transport from cell to intracellular. Ca^2+^ is very important for the growth and development of bones and teeth. It also plays an important role in maintaining the potential difference between the inside and outside of the cell membrane [60]. KCNJ13 is a member of the inward rectifier K^+^ channel (IRK1) superfamily. It is a low-conductance channel that is mainly expressed in the basolateral membrane of epithelial cells, including intestinal epithelial cells, thyroid follicular cells, and proximal and distal convoluted tubular epithelial cells [61]. In this study, PEDV infection could not significantly decrease the mRNA levels of *TRPV6* and *KCNJ13*. These results revealed that PEDV infection did not damage Ca^2+^ and K^+^ transmembrane transport channels in the liver, lung, spleen, lymph node, or gastrocnemius in piglets.

A low concentration of IL-1β mainly exerts an immunomodulatory effect, whereas a high concentration of IL-1β mainly stimulates the expression of inflammation and autoimmune disease-related genes, leading to fever and cachexia. IL-4 is a multifunctional lymphokine that can activate the proliferation of B cells and T cells, and can also play a role in humoral immunity. The most important function of IL-4 is to inhibit inflammation. IL-10 is also an inhibitor of cytokine synthesis. Many studies have confirmed that IL-4 and IL-10 can inhibit monocyte macrophages from producing IL-1β; furthermore, they inhibit the synthesis of cytokines, the production of nitric oxide, and the expression of other costimulatory factors [11,20,38]. TNF-α is a pro-inflammatory cytokine produced by activated macrophages, lymph node cells, and other immune cells when pathogens invade the body [62]. TNF-α can inhibit tumor cell proliferation, induce tumor cell apoptosis, participate in the immune regulation of the body, and induce the expression of related cytokines and their receptor genes in mammals; TNF-α plays an important role in resisting bacterial, viral, and parasitic infections [63]. Some studies have demonstrated that S100A12 can bind to the cell surface through advanced glycation end product receptor and endogenous Toll-like receptor 4, and further activate the intracellular regulatory kinase 1/2 (ERK1/2) and phosphatidylinositol 3-kinase (PI-3K)/AKT signaling pathways in lymph node cells, neutrophils, and monocytes, which induces the production of cytokines such as IL-1β and TNF-a, and promotes the progression of inflammation [37,50,51]. In this study, PEDV infection significantly decreased the mRNA levels of *IL-1β* (spleen, lymph node, and gastrocnemius), *IL-4* (lymph node), *IL-10* (spleen and gastrocnemius), *TNF-α* (liver and spleen), and *S100A12* (liver and spleen). NAC administration decreased the reductions in *IL-4* (lymph node), *IL-10* (spleen), *TNF-α* (spleen), and *S100A12* (spleen) in PEDV-infected piglets. Studies have shown that the moderate inflammation response is beneficial to the body. These results reveal that PEDV infection significantly decreased the inflammatory response levels of the tissues and organs in piglets, and NAC administration improved the inflammatory response capacities of the spleen and lymph nodes by regulating the expression levels of inflammation-related genes.

MMP3 is a protease produced by synovial fibroblasts and chondrocytes in joints. Studies have reported that there is a positive correlation between disease and MMP3 expression, and the severity of rheumatoid arthritis will change with changes in serum MMP3 expression [64]. MMP13 is the main enzyme that targets cartilage degradation. Compared with other MMPs, the expression level of MMP13 is more confined to connective tissue. MMP13 can not only degrade type II collagen in cartilage, but also proteoglycan, type IV and type IX collagen, osteonectin, and basement membrane proteoglycan [65]. TGF-β has multiple biological activities. In addition to regulating development, proliferation, and the immune response, it also plays an important role in the process of tumorigenesis: it can exert a tumor-suppressive effect by inducing apoptosis and cell-cycle arrest [66]. GJA1 is a highly phosphorylated protein that undergoes multiple processing. Both the phosphorylation and the dephosphorylation of GJA1 affect the permeability of gap-junction channels. GJA1 can not only transmit regulatory signals for maintaining the homeostasis of the animal’s environment, but also regulate the normal rhythmic contraction of muscle cells [67]. REG3G, as a secreted protein, is a calcium-dependent phytohemagglutinin, which has the functions of trophic; anti-inflammatory; anti-apoptosis. There is also research showing that REG is mainly related to the occurrence of pancreatitis, diabetes, colitis, and other diseases. In this study, PEDV infection significantly decreased the mRNA levels of *MMP3* (spleen), *MMP13* (spleen), *TGF-β* (spleen, lymph nodes, and gastrocnemius), and *GJA1* (spleen). NAC administration decreased the reductions in the mRNA levels of *MMP3* (spleen), *MMP13* (spleen), *TGF-β* (spleen and lymph node), and *GJA1* (spleen). These results revealed that NAC administration improved the expression levels of *MMP3*, *MMP13*, *TGF-β*, and *GJA1* in the spleens and lymph nodes of PEDV-infected piglets.

## 5. Conclusions

In summary, PEDV infection has the most obvious effects on the redox and functional gene expression levels in the spleens of piglets. The activities of two antioxidant enzymes were significantly weakened, and the mRNA levels of 16 functional genes were significantly down-regulated. However, NAC administration significantly improved the antioxidant level, inflammation level, immune response, and tissue repair ability of the spleen by decreasing the reductions in activities of the two antioxidant enzymes and the mRNA levels of 11 functional genes in the PEDV-infected piglets. In addition, NAC administration improved the antioxidant level, inflammation level, and tissue repair ability of lymph nodes by decreasing the reductions in the activity of SOD and the mRNA levels of IL-1β and TGF-β in PEDV-infected piglets. NAC administration also improved the antioxidant level in the gastrocnemii by decreasing the reduction in the activity of GSH-Px in PEDV-infected piglets. In short, NAC administration not only alleviated intestinal injury, but also improved the redox and functional gene expression levels in the spleen, lymph nodes, and gastrocnemius in PEDV-infected piglets.

## Figures and Tables

**Table 1:** Nutritional levels of the milk replacer (%).

Items	Crude Protein	Crude Fat	Crude Ash	Crude Fiber	Water	Lysine	Calcium	Total Phosphorus
Milk replacer	≥20.0	≥10.0	≥9.0	≥0.3	≤10.0	≥1.4	0.4~1.1	≥0.3

**Table 2:** Sequence of the primers used for qPCR analysis.

Gene	Forward (5′-3′)	Reverse (5′-3′)
*RPL4*	5′-GAGAAACCGTCGCCGAAT-3′	5′-GCCCACCAGGAGCAAGTT-3′
*Keap1*	5′-ACGACGTGGAGACAGAAACGT-3′	5′-GCTTCGCCGATGCTTCA-3′
*Nrf2*	5′-ATCACCTCTTCTGCACCGAA-3′	5′-GCTTTCTCCCGCTCTTTCTG-3′
*GSTO2*	5′-GCCTTGAGATGTGGGAGAGAA-3′	5′-AAGATGGTGTTCTGATAGCCAAGA-3′
*HMOX1*	5′-TTTCTGAGCCTCCAAACACC-3′	5′-AACAAGACGGAAACACGAGACA-3′
*MMP3*	5′-GATGTTGGTTACTTCAGCAC-3′	5′-ATCATTATGTCAGCCTCTCC-3′
*MMP13*	5′-AGTTTGGCCATTCCTTAGGTCTTG-3′	5′-GGCTTTTGCCAGTGTAGGTATAGAT-3′
*TGFβ*	5′-TGGAAAGCGGCAACCAA-3′	5′-GCCCGAGAGAGCAATACAGG-3′
*REG3G*	5′-CTGTCTCAGGTCCAAGGTGAAG-3′	5′-CAAGGCATAGCAGTAGGAAGCA-3′
*GJA1*	5′-CAAATCCTTCCCCATCTCTCAC-3′	5′-TCAGTTTCTCTTCCTTTCGCATC-3′
*IL-1β*	5′-AGGCGATTATTAAATG-3′	5′-TTTTTGCATCCGTCAATGACA-3′
*IL-4*	5′-TACCAGCAACTTCGTCCAC-3′	5′-ATCGTCTTTAGCCTTTCCAA-3′
*IL-6*	5′-TACTGGCAGAAACAACCTG-3′	5′-GTACTAATCTGCACAGCCTC-3′
*IL-10*	5′-CGGCGCTGTCATCAATTTCTG-3′	5′-CCCCTCTCTTGGAGCTTGCTA-3′
*TNF-α*	5′-TCCAATGCAGAGTGGTATG-3′	5′-AGTTTACTCTCAC-3′
*S100A12*	5′-TTGAAGGGTGAACGCAAGG-3′	5′-AATGCCCCAACCGAACTG-3′
*TRPV6*	5′-AGGAGCTGGTGAGCCTCAAGT-3′	5′-GGGGTCAGTTTGGTTGTTGG-3′
*KCNJ13*	5′-CCCAAATCAGAGCATTCCATTCA-3′	5′-AAGTATGGTGTGGTGGCCGGTT-3′
*IFN-α*	5′-TGCCATAAGGGTGTCAGGTATTT-3′	5′-TGCTTTGCTCTTGCCCTCTAC-3′
*MX1*	5′-AGTGCGGCTGTTTACCAAG-3′	5′-TTCACAAACCCTGGCAACTC-3′
*OASL*	5′-GGCACCCCTGTTTTCCTCT-3′	5′-AGCACCGCTTTTGGATGG-3′
*ISG15*	5′-AGCATGGTCCTGTTGATGGTG-3′	5′-CAGAAATGGTCAGCTTGCACG-3′
*GBP2*	5′-ACCAGGAGGTTTTCGTCTCTCTATT-3′	5′-TCCTCTGCCTGTATCCCCTTT-3′

**Table 3:** Effects of NAC administration on redox status in piglets infected with PEDV.

Items	−PEDV		+PEDV		SEM	*p*-Value		
	−NAC	+NAC	−NAC	+NAC		PEDV	NAC	PEDV × NAC
CAT (U/mg prot)								
Gastrocnemius	7.28 ^a^	9.43 ^b^	9.26 ^b^	12.73 ^c^	0.82	<0.001	<0.001	<0.001
Lung	25.15	23.68	12.34	14.89	1.99	<0.001	0.71	0.16
Lymph node	16.43 ^c^	11.39 ^b^	4.42 ^a^	5.09 ^a^	1.53	<0.001	0.07	<0.001
Spleen	13.32 ^a^	20.17 ^b^	28.02 ^c^	19.73 ^b^	2.24	<0.001	0.61	<0.001
Liver	67.35	96.62	58.76	111.59	10.51	0.71	<0.001	0.18
MPO (U/g prot)								
Gastrocnemius	0.16	0.18	0.21	0.21	0.01	0.27	0.09	0.02
Lung	0.35	0.37	0.42	0.41	0.02	0.11	0.94	0.41
Lymph node	0.48 ^a^	0.59 ^a^	0.643 ^a^	1.35 ^b^	0.12	<0.001	<0.001	<0.001
Spleen	11.29 ^b^	1.06 ^a^	1.77 ^a^	1.61 ^a^	1.34	<0.001	<0.001	<0.001
Liver	0.22	0.26	0.19	0.22	0.02	0.10	0.15	0.61
SOD (U/mg prot)								
Gastrocnemius	216.86	207.29	212.46	207.03	8.13	0.35	0.772	0.79
Lung	101.87	93.77	105.88	102.89	4.85	0.18	0.26	0.61
Lymph node	184.86 ^c^	145.35 ^ab^	135.30 ^a^	173.16 ^bc^	12.48	0.271	0.93	<0.001
Spleen	152.96	148.81	116.01	83.95	12.01	<0.001	0.04	0.11
Liver	57.49	61.02	46.89	58.85	4.71	0.21	0.13	0.41
GSH-Px (U/mg prot)								
Gastrocnemius	0.09 ^b^	0.05 ^a^	0.05 ^a^	0.10 ^b^	0.01	0.2	0.62	<0.001
Lung	91.46	91.66	114.19	127.05	7.28	<0.001	0.31	0.32
Lymph node	207.401 ^ab^	178.67 ^a^	198.04 ^ab^	231.20 ^b^	14.08	0.07	0.85	0.01
Spleen	229.07	187.69	128.6	126.71	16.35	<0.001	0.05	0.07
Liver	22.14	20.89	22.34	23.91	2.06	0.41	0.93	0.47
H_2_O_2_ (mol/mg prot)								
Gastrocnemius	15.99	14.36	13.94	15.51	1.33	0.71	0.97	0.19
Lung	45.31 ^a^	43.83 ^a^	47.95 ^a^	29.82 ^b^	3.97	0.01	0.11	0.02
Lymph node	3.36 ^ab^	3.48 ^bc^	2.64 ^a^	4.27 ^c^	0.34	0.89	0.01	0.01
Spleen	85.92	76.55	49.83	62.31	7.01	<0.001	0.77	0.05
Liver	21.23	27.81	22.84	22.16	3.36	0.51	0.33	0.23
MDA (nmol/mg prot)								
Gastrocnemius	0.61 ^a^	0.51 ^a^	0.66 ^a^	1.42 ^b^	0.13	<0.001	<0.001	<0.001
Lung	3.21	2.26	2.35	1.36	0.22	<0.001	<0.001	0.91
Lymph node	2.73	2.13	2.11	1.85	0.19	0.01	0.01	0.31
Spleen	4.15	3.84	4.78	4.17	0.34	0.127	0.15	0.62
Liver	0.98	1.17	2.11	1.23	0.19	0.05	0.11	0.16

Values are mean and pooled SEM, n = 8; CAT = catalase; MPO = myeloperoxidase; GSH-Px = glutathione peroxidase; H_2_O_2_ = hydrogen peroxide; MDA = malondialdehyde; ^a,b,c^ Within a row, means with different superscripts differ, *p* < 0.05.

**Table 4:** Effects of NAC administration on the mRNA expression of *Keap1*, *Nrf2*, *GSTO2*, and *HMOX1* in the liver, spleen, lung, lymph node, and gastrocnemius muscle in piglets infected with PEDV.

Items	−PEDV		+PEDV		SEM	*p*-Value		
	−NAC	+NAC	−NAC	+NAC		PEDV	NAC	PEDV × NAC
*Keap1*								
Liver	1.00 ^b^	1.81 ^a^	1.05 ^b^	0.88 ^b^	0.25	0.09	0.20	0.06
Lung	1.00 ^b^	0.82 ^b^	0.88 ^b^	1.38 ^a^	0.06	0.01	0.01	<0.001
Lymph node	1.00	0.83	1.22	1.29	0.08	<0.001	0.55	0.17
Gastrocnemius	1.00	0.99	0.88	1.14	0.07	0.81	0.11	0.11
Spleen	1.00 ^b^	0.95 ^bc^	0.77 ^c^	1.39 ^a^	0.07	0.17	0.01	<0.001
*Nrf2*								
Liver	1.00 ^b^	2.01 ^a^	0.82 ^b^	0.53 ^c^	0.08	<0.001	<0.001	<0.001
Lung	1.00 ^c^	1.55 ^a^	1.29 ^ab^	1.07 ^bc^	0.09	0.33	0.09	<0.001
Lymph node	1.00	0.86	0.83	0.70	0.06	0.01	0.04	0.95
Gastrocnemius	1.00 ^c^	1.16 ^c^	3.40 ^a^	1.77 ^b^	0.15	<0.001	<0.001	<0.001
Spleen	1.00 ^b^	0.24 ^d^	0.47 ^c^	1.35 ^a^	0.05	<0.001	0.27	<0.001
*GSTO2*								
Liver	1.00 ^a^	0.42 ^b^	0.32 ^bc^	0.25 ^c^	0.04	<0.001	<0.001	<0.001
Lung	1.00 ^b^	1.44 ^a^	1.14 ^b^	1.06 ^b^	0.08	0.18	0.03	0.01
Lymph node	1.00 ^a^	0.21 ^bc^	0.25 ^b^	0.14 ^c^	0.03	<0.001	<0.001	<0.001
Gastrocnemius	1.00 ^a^	0.16 ^c^	0.28 ^b^	0.26 ^b^	0.02	<0.001	<0.001	<0.001
Spleen	1.00 ^a^	0.19 ^b^	0.23 ^b^	0.16 ^b^	0.03	<0.001	<0.001	<0.001
*HMOX1*								
Liver	1.00 ^b^	1.42 ^a^	0.78 ^c^	0.86 ^bc^	0.06	<0.001	<0.001	0.01
Lung	1.00 ^a^	0.72 ^b^	0.81 ^ab^	0.96 ^a^	0.06	0.71	0.35	0.01
Lymph node	1.00	0.73	1.75	1.15	0.09	<0.001	<0.001	0.09
Gastrocnemius	1.00	0.42	0.85	0.50	0.05	0.58	<0.001	0.06
Spleen	1.00 ^a^	0.73 ^c^	0.72 ^c^	1.49 ^b^	0.08	0.01	0.01	<0.001

Values are mean and pooled SEM, n = 8; ^a,b,c,d^ within a row, means with different superscripts differ, *p* < 0.05.

**Table 5:** Effects of NAC administration on mRNA of *IFN-α*, *MX1*, *OASL*, and *ISG15* in the liver, spleen, lung, lymph node, and gastrocnemius muscle in piglets infected with PEDV.

Items	−PEDV		+PEDV		SEM	*p*-Value		
	−NAC	+NAC	−NAC	+NAC		PEDV	NAC	PEDV × NAC
*IFN-α*								
Liver	1.00 ^b^	1.53 ^a^	0.75 ^c^	0.54 ^c^	0.08	<0.001	0.07	<0.001
Spleen	1.00 ^a^	0.59 ^c^	0.80 ^b^	1.16 ^a^	0.06	<0.001	0.67	<0.001
Lymph node	1.00 ^d^	2.59 ^a^	1.66 ^c^	2.07 ^b^	0.12	0.55	<0.001	<0.001
Gastrocnemius	1.00	1.03	1.33	1.25	0.09	0.01	0.75	0.54
*MX1*								
Liver	1.00 ^b^	1.63 ^a^	1.59 ^a^	1.56 ^a^	0.10	0.02	0.01	<0.001
Spleen	1.00 ^a^	0.87 ^a^	0.51 ^b^	0.94 ^a^	0.05	<0.001	0.01	<0.001
Lymph node	1.00	1.55	1.64	2.24	0.12	<0.001	<0.001	0.83
Gastrocnemius	1.00 ^c^	1.28 ^bc^	1.80 ^a^	1.39 ^b^	0.11	<0.001	0.56	0.01
*OASL*								
Liver	1.00	1.42	1.30	1.52	0.28	0.50	0.28	0.73
Spleen	1.00 ^a^	0.63 ^b^	0.60 ^b^	1.07 ^a^	0.07	0.78	0.50	<0.001
Lymph nod	1.00 ^c^	0.92 ^c^	2.53 ^a^	1.74 ^b^	0.13	<0.001	<0.001	0.01
Gastrocnemius	1.00 ^b^	0.84 ^b^	2.27 ^a^	0.76 ^b^	0.11	<0.001	<0.001	<0.001
*ISG15*								
Liver	1.00	0.98	2.72	2.42	0.13	<0.001	0.26	0.31
Spleen	1.00 ^c^	1.94 ^a^	1.05 ^c^	1.44 ^b^	0.10	0.03	<0.001	0.01
Lymph node	1.00 ^c^	0.91 ^c^	1.74 ^b^	2.35 ^a^	0.11	<0.001	0.03	<0.001
Gastrocnemius	1.00 ^b^	1.17 ^b^	1.85 ^a^	1.30 ^b^	0.10	<0.001	0.06	<0.001

Values are mean and pooled SEM, n = 8; ^a,b,c,d^ within a row, means with different superscripts differ, *p* < 0.05.

**Table 6:** Effects of NAC administration on mRNA expression levels of *TRPV6* and *KCNJ13* in the liver, spleen, lung, lymph node, and gastrocnemius muscle in piglets infected with PEDV.

Items	−PEDV		+PEDV		SEM	*p*-Value		
	−NAC	+NAC	−NAC	+NAC		PEDV	NAC	PEDV × NAC
*TRPV6*								
Liver	1.00 ^b^	1.27 ^a^	0.90 ^b^	0.56 ^c^	0.07	<0.001	0.60	<0.001
Lung	1.00 ^b^	1.59 ^a^	1.02 ^b^	1.01 ^b^	0.08	<0.001	<0.001	<0.001
Spleen	1.00	1.04	0.85	0.95	0.08	0.14	0.42	0.71
Lymph node	1.00	1.49	1.17	1.34	0.10	0.91	<0.001	0.13
Gastrocnemius	1.00	1.04	1.85	2.06	0.12	<0.001	0.28	0.46
*KCNJ13*								
Liver	1.00 ^b^	1.83 ^a^	1.15 ^b^	0.62 ^c^	0.08	<0.001	0.07	<0.001
Lung	1.00	1.03	1.16	0.97	0.08	0.51	0.30	0.16
Spleen	1.00	1.11	1.17	1.50	0.09	<0.001	0.02	0.23
Lymph node	1.00	1.52	1.39	1.70	0.09	<0.001	<0.001	0.26
Gastrocnemius	1.00 ^b^	0.94 ^b^	0.99 ^b^	1.38 ^a^	0.09	0.02	0.07	0.02

Values are mean and pooled SEM, n = 8; ^a,b,c^ within a row, means with different superscripts differ, *p* < 0.05.

**Table 7:** Effects of NAC administration on the mRNA expression of *IL-1β*, *IL-4*, *IL-10*, *TNF-α*, and *S100A12* in liver, spleen, lung, lymph node, and gastrocnemius muscle in piglets infected with PEDV.

Items	−PEDV		+PEDV		SEM	*p*-Value		
	−NAC	+NAC	−NAC	+NAC		PEDV	NAC	PEDV × NAC
*IL-1β*								
Liver	1.00 ^b^	1.34 ^a^	0.90 ^b^	0.80 ^b^	0.07	<0.001	0.12	0.01
Spleen	1.00 ^a^	0.68 ^b^	0.73 ^b^	0.79 ^b^	0.06	0.14	0.03	<0.001
Lymph node	1.00 ^b^	1.54 ^a^	0.67 ^c^	0.53 ^c^	0.07	<0.001	0.01	<0.001
Gastrocnemius	1.00 ^a^	0.64 ^b^	0.59 ^b^	0.72 ^b^	0.05	<0.001	0.02	<0.001
*IL-4*								
Lung	1.00 ^b^	1.61 ^a^	0.90 ^b^	1.05 ^b^	0.06	<0.001	<0.001	<0.001
Lymph node	1.00 ^a^	0.79 ^b^	0.18 ^d^	0.48 ^c^	0.05	<0.001	0.41	<0.001
*IL-10*								
Liver	1.00	1.24	0.85	0.89	0.08	<0.001	0.09	0.23
Lung	1.00	1.20	0.85	1.01	0.08	0.04	0.03	0.78
Spleen	1.00 ^a^	0.75 ^b^	0.41 ^c^	0.87 ^ab^	0.06	<0.001	0.10	<0.001
Lymph node	1.00	0.92	1.46	1.21	0.09	<0.001	0.08	0.36
Gastrocnemius	1.00 ^a^	0.68 ^b^	0.76 ^b^	0.83 ^ab^	0.06	0.46	0.06	<0.001
*TNF-α*								
Liver	1.00 ^b^	1.28 ^a^	0.57 ^c^	0.59 ^c^	0.06	<0.001	0.02	0.05
Lung	1.00 ^ab^	1.21 ^a^	1.07 ^ab^	0.95 ^b^	0.08	0.27	0.59	0.05
Spleen	1.00 ^a^	0.46 ^b^	0.41 ^b^	1.01 ^a^	0.06	0.72	0.61	<0.001
Lymph node	1.00	1.24	0.91	1.04	0.08	0.07	0.02	0.47
Gastrocnemius	1.00	0.82	0.79	0.61	0.06	<0.001	0.01	0.98
*S100A12*								
Liver	1.00 ^b^	1.39 ^a^	1.39 ^a^	1.11 ^b^	0.09	0.54	0.57	<0.001
Lung	1.00 ^b^	1.38 ^a^	1.11 ^b^	1.09 ^b^	0.08	0.27	0.03	0.02
Spleen	1.00 ^a^	0.68 ^b^	0.54 ^b^	0.97 ^a^	0.06	0.19	0.40	<0.001
Lymph node	1.00	0.67	1.61	0.91	0.09	<0.001	<0.001	0.04
Gastrocnemius	1.00	0.63	0.90	0.60	0.06	0.30	<0.001	0.60

Values are mean and pooled SEM, n = 8; ^a,b,c,d^ Within a row, means with different superscripts differ, *p* < 0.05.

**Table 8:** Effects of NAC administration on mRNA expression of *MMP3*, *MMP13*, *REG3G*, *TGF-β*, and *GJA1* in liver, spleen, lung, lymph node, and gastrocnemius muscle in piglets infected with PEDV.

Items	−PEDV		+PEDV		SEM	*p*-Value		
	−NAC	+NAC	−NAC	+NAC		PEDV	NAC	PEDV × NAC
*MMP3*								
Lung	1.00	1.15	2.37	2.17	0.13	<0.001	0.88	0.21
Spleen	1.00 ^b^	0.39 ^c^	0.33 ^c^	3.13 ^a^	0.13	<0.001	<0.001	<0.001
Lymph node	1.00 ^c^	1.63 ^b^	2.68 ^a^	1.54 ^b^	0.15	<0.001	0.09	<0.001
Gastrocnemius	1.00 ^bc^	1.60 ^a^	1.18 ^b^	0.89 ^c^	0.09	0.01	0.09	<0.001
*MMP13*								
Liver	1.00 ^b^	1.45 ^a^	1.54 ^a^	1.35 ^a^	0.10	0.05	0.23	<0.001
Lung	1.00	1.22	1.97	1.93	0.11	<0.001	0.43	0.26
Spleen	1.00 ^a^	0.87 ^a^	0.58 ^b^	0.99 ^a^	0.07	0.03	0.04	<0.001
Lymph node	1.00 ^c^	1.45 ^a^	1.30 ^ab^	1.17 ^bc^	0.08	0.93	0.04	<0.001
Gastrocnemius	1.00 ^b^	0.84 ^b^	0.91 ^b^	1.59 ^a^	0.08	<0.001	<0.001	<0.001
*REG3G*								
Spleen	1.00 ^c^	1.47 ^b^	1.75 ^b^	3.72 ^a^	0.16	<0.001	<0.001	<0.001
Gastrocnemius	1.00 ^c^	1.07 ^bc^	1.63 ^a^	1.29 ^b^	0.09	<0.001	0.13	0.03
*TGF-β*								
Liver	1.00	1.26	0.63	0.82	0.08	<0.001	0.01	0.63
Lung	1.00	0.87	0.98	0.81	0.06	0.52	0.02	0.70
Spleen	1.00 ^a^	0.75 ^b^	0.71 ^b^	0.98 ^a^	0.06	0.65	0.91	<0.001
Lymph node	1.00 ^a^	0.95 ^ab^	0.77 ^b^	1.01 ^a^	0.07	0.24	0.18	0.04
Gastrocnemius	1.00 ^a^	0.72 ^b^	0.71 ^b^	0.71 ^b^	0.04	<0.001	<0.001	<0.001
*GJA1*								
Liver	1.00 ^b^	1.49 ^a^	1.30 ^a^	0.91 ^b^	0.09	0.15	0.61	<0.001
Lung	1.00	1.05	0.82	0.86	0.08	0.02	0.55	0.90
Spleen	1.00 ^b^	0.93 ^b^	0.66 ^c^	1.17 ^a^	0.06	0.37	<0.001	<0.001
Lymph node	1.00	1.11	1.15	1.16	0.07	0.19	0.41	0.48
Gastrocnemius	1.00	1.33	0.88	1.10	0.08	0.03	<0.001	0.47

Values are mean and pooled SEM, n = 8; ^a,b,c^ Within a row, means with different superscripts differ, *p* < 0.05.

## Data Availability

The datasets used and/or analyzed during the current study are available from the corresponding author on reasonable request.
